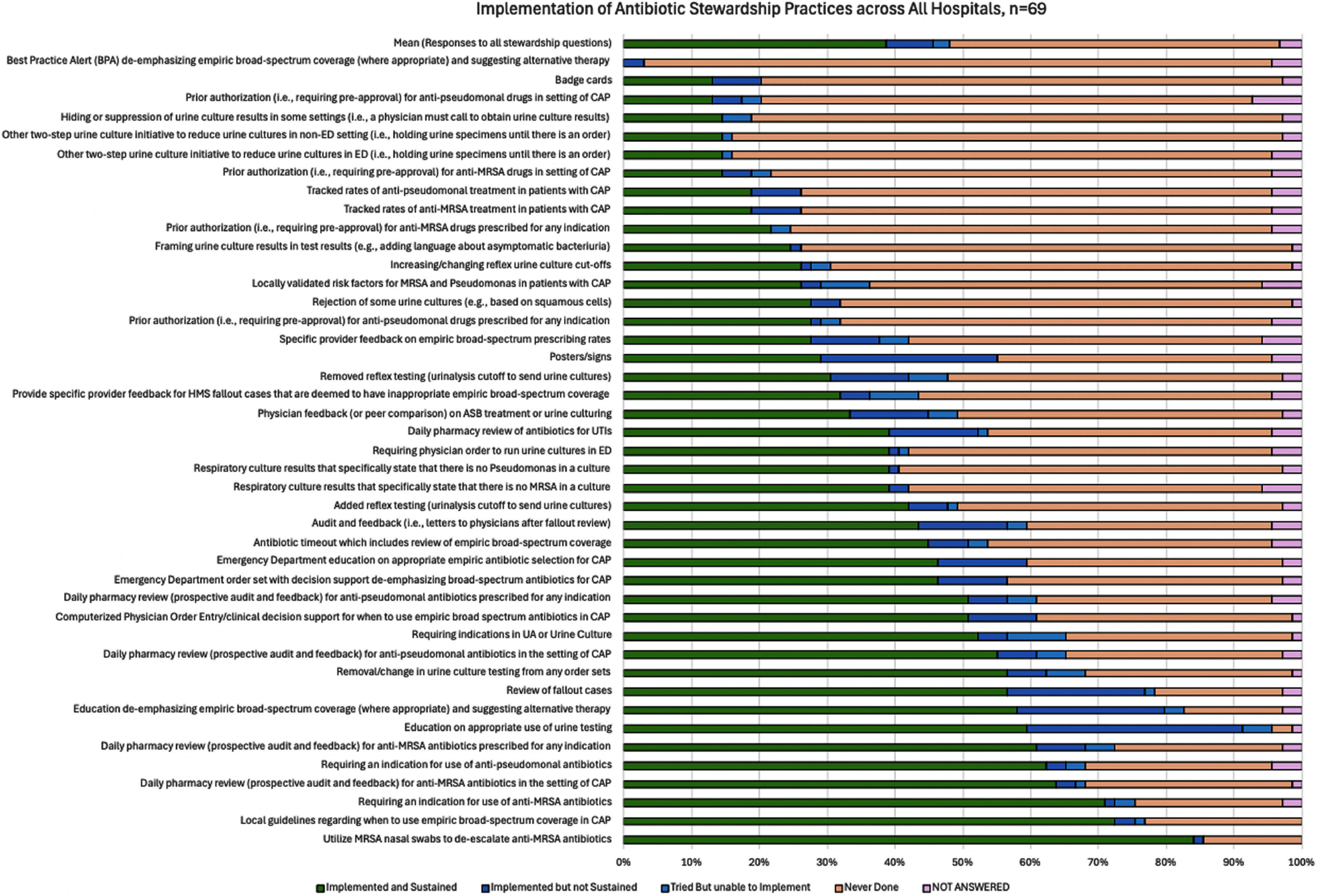# Relationship between Hospital Characteristics and Reported Feasibility and Implementation of Antibiotic Stewardship Interventions

**DOI:** 10.1017/ash.2025.216

**Published:** 2025-09-24

**Authors:** Grant Gosden, Julie Szymczak, Tejal Gandhi, Lindsay Petty, Robert Neetz, Ashwin Gupta, Jennifer Horowitz, Elizabeth McLaughlin, James Harrison, Anurag Malani, Mariam Younas, Scott Flanders, Valerie Vaughn

**Affiliations:** 1 University of Utah; 2 University of Utah School of Medicine; 3 University of Michigan Medical School; 4 Michigan Medicine; 5 MyMichigan Health; 6 University of Michgian/VA Ann Arbor Healthcare System; 7 DIvision of Hospital Medicine, Michigan Medicine; 8 University of Michgian; 9 Division of Hospital Medicine, University of California San Francisco; 10 Trinity Health Michigan; 11 Michigan State University - College of Human Medicine, Hurley Medical Center

## Abstract

**Background:** Hospital antibiotic stewardship programs (ASPs) are essential for reducing unnecessary antibiotic use and combating antimicrobial resistance. While many ASP interventions have been described, their feasibility and sustainability remain unclear, particularly for smaller hospitals with limited informatics resources. This study aimed to assess the feasibility and sustainability of common ASP interventions and examine the impact of hospital bed size on sustainability. **Methods:** A cross-sectional survey was conducted between April and May 2023 across 69 hospitals in Michigan participating in the Michigan Hospital Medicine Safety Consortium, representing both large (<200 beds) hospitals. Quality improvement or antimicrobial stewardship staff from each hospital ranked the feasibility of 7 common antibiotic stewardship interventions on a scale from 1 (easiest) to 7 (hardest). Respondents were then asked to report their status with 43 individual stewardship interventions as: a) implemented and sustained, b) implemented, but not sustained, c) tried but unable to implement, or d) never done. We used descriptive statistics and Fisher’s exact tests to compare reported intervention feasibility and implementation by hospital bed size (small vs. large). **Results:** All 69 hospitals responded to our survey (100% response rate). Across all hospitals, increasing audit and feedback by pharmacists was reported as the easiest new intervention to implement, whereas starting clinician peer comparison was reported as the hardest (Figure 1). Hospitals had implemented and sustained multiple stewardship interventions with substantial variation by intervention (Figure 2). Reported feasibility of the 7 common stewardship interventions did not significantly differ between large and small hospitals. However, small hospitals had significantly higher implementation of five antibiotic stewardship interventions: removal or change in order sets in urine culture testing (implemented by 73.1% of small hospitals vs. 46.3% of large hospitals; p=0.04), two-step urine culture initiative to reduce unnecessary testing (27% vs. 7%; p=0.04), Emergency Department order set with decision support de-emphasizing broad-spectrum antibiotics for CAP (77% vs. 48%; p = 0.02), daily pharmacy review of antibiotics for UTIs (58% vs. 30%; p=0.04), and daily pharmacy review of anti-pseudomonal antibiotics for CAP (73% vs. 46%; p=0.04). **Conclusions:** Feasibility and implementation of ASP interventions varied widely, with most interventions sustained once implemented. Technical solutions were 26.4% more likely to be sustained than adaptive ones. Small hospitals showed higher implementation rates for several interventions, potentially due to smaller patient populations and fewer administrative barriers. Hospitals should tailor ASP priorities to their local context, focusing on feasible and sustainable interventions.

**Figure:**
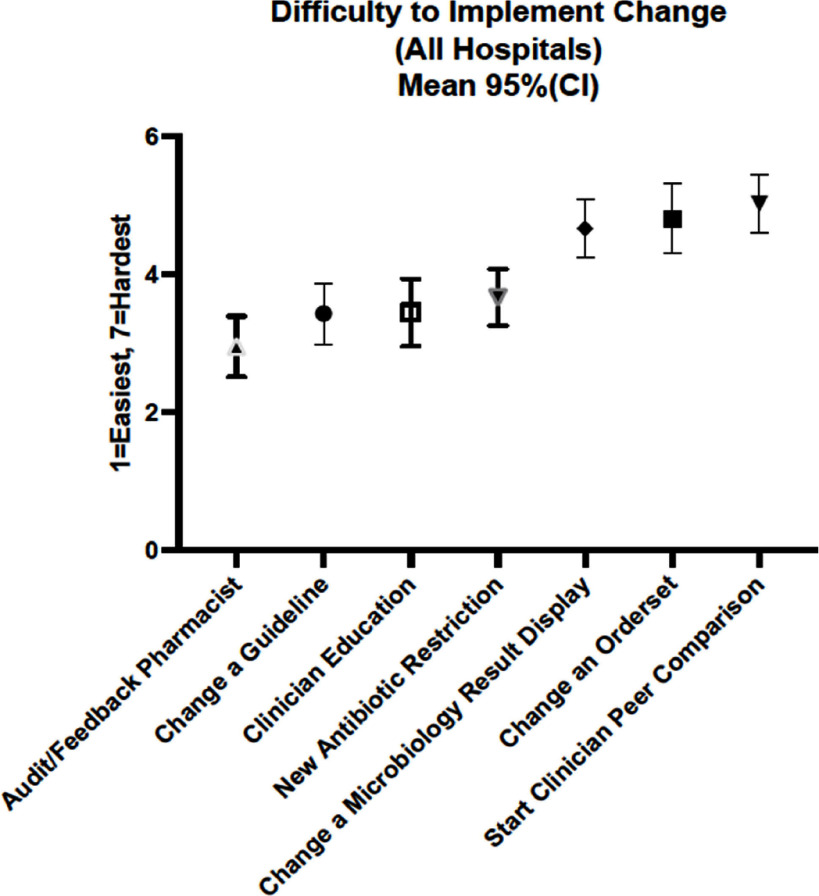

**Figure:**